# Breeding for reduced seed dormancy to domesticate new grass species

**DOI:** 10.1590/1678-4685-GMB-2023-0262

**Published:** 2024-04-12

**Authors:** Nicolás Glison, Paola Gaiero, Eliana Monteverde, Pablo R. Speranza

**Affiliations:** 1 Universidad de la República, Facultad de Agronomía, Departamento de Biología Vegetal, Montevideo, Uruguay.; 2 University of Illinois, Department of Crop Sciences, Urbana, IL, USA.

**Keywords:** Domestication syndrome, crop establishment, forage breeding, ABA-GA hormonal balance, warm-season forage grasses

## Abstract

Introducing new grass species into cultivation has long been proposed as beneficial to increase the sustainability and diversity of productive systems. However, wild species with potential tend to show high seed dormancy, causing slow, poor, and unsynchronized seedling emergence. Meanwhile, domesticated species, such as cereals, show lower seed dormancy, facilitating their successful establishment. In this work, we conduct a review of phenotypic variation on seed dormancy and its genetic and molecular basis. This quantitative and highly heritable trait shows phenotype plasticity which is modulated by environmental factors. The level of dormancy depends on the expression of genes associated with the metabolism and sensitivity to the hormones abscisic acid (ABA) and gibberellins (GA), along with other dormancy-specific genes. The genetic regulation of these traits is highly conserved across species. The low seed dormancy observed in cereals and some temperate forages was mostly unconsciously selected during various domestication processes. Emphasis is placed on selecting materials with low seed dormancy for warm-season forage grasses to improve their establishment and adoption. Finally, we review advances in the domestication of dallisgrass, where seed dormancy was considered a focus trait throughout the process.

## Introduction

An essential part of worldwide grain and forage production depends on a few domesticated and semi-domesticated grass species adapted to agroecosystems and human management (Fuller and Allaby, 2009). Traditional agroecosystems face sustainability problems, such as a high dependence on chemical inputs, effluent pollution by excessive fertilization, negative carbon footprint, erosion, and soil degradation. Crop diversification by increasing the domestication of native species has been proposed as a possible solution, assuming that these species are already adapted to regional climate and soil characteristics (Van Tassel and De Haan, 2013). However, although attractive plant material can be found, cultivars directly derived from wild species often do not reach the seedling emergence rates expected for productive purposes, among other disadvantages. Particularly, warm-season forage grasses usually show low germination rates, strongly limiting their widespread use (Adkins et al., 2002). One of the leading causes of this response is seed dormancy, which is often high in wild and semi-domesticated species but low in full-domesticated species that show high seedling emergence rates, like cereals (Fuller and Allaby, 2009). Seed dormancy is an intrinsic condition of the seed that prevents germination under otherwise adequate environmental conditions (Benech-Arnold et al., 2000). Dormancy is a quantitative trait of seed populations, and its level is related to the amplitude of the range of environmental conditions, such as temperature and moisture, in which a given seed population can germinate (Batlla and Benech-Arnold, 2010). 

This work reviews the characteristics of physiological dormancy, the most prevalent class of seed dormancy found in grasses and many other taxa. Other classes of seed dormancy, such as physical and morphological dormancy, are characterized by seed coat impermeability in the former and underdeveloped embryos in the latter. However, none of these mechanisms are present in grass seeds. We depict the factors contributing to phenotypic variation in physiological dormancy (hereafter referred to as seed dormancy) within and among species and populations, its plasticity, and the key genetic and molecular factors involved. We discuss the differences in seed dormancy between domesticated cereal crops and semi- or non-domesticated forage crops. We highlight the relevance of breeding for reduced seed dormancy as part of the domestication process, especially for warm-season forage grasses, for which fewer studies have been reported compared to cool-season grasses. Finally, we describe the progress made in understanding the phenotype and genetic control of seed dormancy in the domestication of *Paspalum* species of the Dilatata group (dallisgrass). 

## Sources of variation in seed dormancy phenotype

### Intrapopulation variability

Improving the selection of low-dormant genotypes in non-domesticated species requires understanding the sources contributing to seed dormancy variation. The dormancy level of seeds produced by a given genotype can vary greatly within a single season or across different years and locations. Environmental conditions as well as phenological and positional factors that occur during seed development on the mother plant, collectively known as maternal effects (Mitchell et al., 2017), strongly modulate the dormancy level of mature seeds (primary dormancy). This intrapopulation variability within a season allows a bet-hedging strategy where germination may occur in pulses, keeping non-germinated viable seeds in the soil for the next season and increasing the chance of persistence of the species (Donohue et al., 2010). On the other hand, variability across years and locations also holds significant adaptative value under fluctuating climates (Mitchell *et al.*, 2017). It is important to consider the plastic components of variation when phenotyping seed dormancy. This task requires evaluating the trait under different conditions for a single genotype.

**Figure 1 -:**
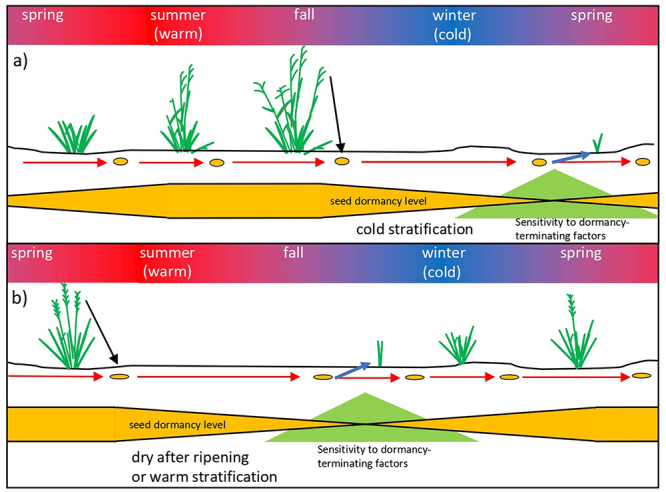
Life cycle of warm- (a) and cool-season (b) grasses throughout a year in temperate regions. The color variations within the top rectangle of each figure represent variations in temperature and seasons: cooler periods are represented in blue, and warmer phases in red. Below the ground in both illustrations, the heights of the orange and green shapes show the changes in seed dormancy level and sensitivity to dormancy-terminating factors (light, nitrate, alternating temperatures) for seeds in soil over a year (red arrows). Each year, the soil seed bank is enriched by seed dispersion (black arrow), followed by a thinning due to the germination season (blue arrows).

Following seed dispersal or harvest, the level of primary dormancy typically decreases. However, it may either remain unchanged or even increase depending mainly on soil temperature, seed moisture, and time. These changes in seed dormancy caused by the environment are known as stratification or dry after-ripening depending on the moisture level during the process (Figure 1); in the former, seeds are hydrated while remaining dry in the latter. The quantitative effects of stratification or dry after-ripening have been well studied in several species and can be modeled using hydro and thermal time models (Batlla and Benech-Arnold, 2010). The interaction between temperature and seed moisture is significant (Malavert et al., 2021), and changes in dormancy in response to these environmental factors and time are heavily influenced by genetic factors.

Achieving low dormancy levels after stratification and dry after-ripening may not be enough to promote seed germination under field conditions. Environmental factors such as light, nitrate, and daily alternating temperatures may be necessary to overcome the final barriers of dormancy (Benech-Arnold et al., 2000). These factors allow the estimation of the depth at which each seed lies in the soil and the density of vegetation above the soil surface. However, the efficiency of breaking dormancy in response to these factors is negatively correlated with seed dormancy (Figure 1). Highly dormant seeds often need more specific daily alternating temperatures or light requirements than seeds with low dormancy (Batlla and Benech-Arnold, 2010). Variability in the response to these factors needs to be taken into account when approaching the *de novo* domestication of grass species.

## Interpopulation variability

Variation among species and populations within a species in seed dormancy phenotypes is expected due to the functional role of this trait in ecological adaptation to the prevailing climatic and topographic factors in the locations where each species or population is found (Donohue et al., 2010). However, seed dormancy often exhibits a strong phylogenetic signature. Species from a particular taxon may show similarities in their dormancy phenotypes, such as the temperature range conducive to germination or the requirements to reduce seed dormancy (Adkins et al., 2002). This information can be helpful when studying seed dormancy in new species.

In temperate and subtropical regions, cold-season and warm-season species can coexist. Usually, cold-season species grow from fall through winter and flower in spring, while warm-season species grow from spring through summer and flower later in the season (Figure 1). The seed dormancy phenotype of each group of species contributes to such life-cycle behavior. Cold stratification efficiently reduces seed dormancy in warm-season species but may increase seed dormancy for cool-season species, while warm stratification usually has the opposite effect (Batlla and Benech-Arnold, 2010) (Figure 1). Consequently, germination and seedling emergence are more likely around the beginning of the growing season for each group of species. This dormancy cycle is essential for annual plants to ensure that most seedling emergence occurs in the right season, but it is also crucial for the regeneration of perennial species.

## Genetic and molecular basis of seed dormancy variation

Seed dormancy is a quantitative trait, and its phenotypic expression is influenced by multiple loci across the genome. Environmental factors during seed development (maternal effects) and after dispersion strongly modulate the expression of some dormancy genes, contributing to the high plasticity of this trait (Graeber et al., 2012; Finch-Savage and Footitt, 2017). Despite this, seed dormancy is affected by some major genes that are responsible for the high heritability that dormancy often exhibits. Seed dormancy is primarily controlled by additive effects (Bentsink et al., 2010), but dominance and epistatic effects should not be overlooked. This section briefly describes the main framework for genetic control of physiological dormancy developed in model species (like *Arabidopsis thaliana* L. and cereal crops). Homologous seed dormancy genes were found in several other species, suggesting that the general mechanism of physiological dormancy is highly conserved among plant species (Graeber *et al.*, 2012; Subburaj et al., 2016; Carrillo-Barral et al., 2020). 

**Figure 2 -:**
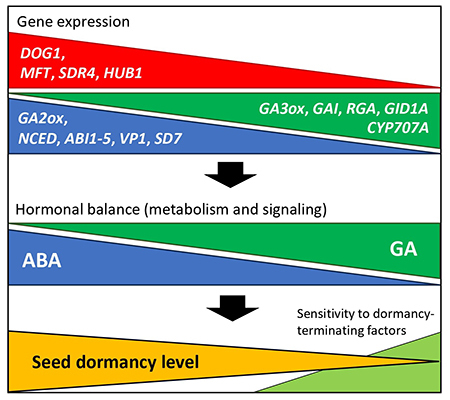
Dynamic expression of key dormancy genes and the abscisic acid-gibberellins (ABA-GA) hormonal balance according to variation in seed dormancy level. The extent of gene expression, ABA-GA balance, and dormancy levels is represented by the size of each triangle at any vertical section. Upper triangles: seed dormancy-specific genes (*DOG1*, *MFT*, *SDR4*) and chromatin remodeling genes (*HUB1*) in red, GA synthesis (*GA3ox*) and signaling (*GAI*, *RGA*, *GID1A*) and ABA degradation genes (*CYP707A*) in green, and ABA synthesis (*NCED*) and signaling (*ABI1-5*, *VP1*, *SD7*) and GA degradation genes (*GA2ox*) in blue. The ABA-GA hormonal balance (middle blue and green triangles) results from gene expression and determines seed dormancy level and consequent sensitivity to dormancy-terminating factors (lower orange and light green triangles, respectively). Gene names: *DELAY OF GERMINATION 1* (*DOG1*), *MOTHER OF FT AND TFL 1* (*MFT*), *SEED DORMANCY 4* (*SDR4*), *HISTONE MONOUBIQUITINATION 1* (*HUB1*), *GA3 OXIDASE* (*GA3ox*), *GIBBERELIN INSENSITIVE* (*GAI*), *REPRESSOR OF GAI* (*RGA*), *GIBBERELIN INSENITIVE DWARF 1* (*GID1A*, a GA receptor), *CYTOCHROME P450 707A* (*CYP707A*), *NINE-CIS-EPOXYCAROTENOID DIOXYGENASE* (*NCED*), *ABA INSENSITIVE 1-5* (*ABI1-5*), *VIVIPAROUS 1* (*VP1*), *SEED DORMANCY 7* (*SD7*), *GA2 OXIDASE* (*GA2ox*).

The expression of genes related to the metabolism and sensitivity to abscisic acid (ABA) and gibberellins (GA) plays a central role in seed dormancy phenotype, although other hormonal groups such as auxins, ethylene, and karrikins also affect this trait (Graeber et al., 2012). When seeds with high dormancy levels undergo initial water uptake (imbibition), they actively accumulate *de novo* ABA while active GA levels are reduced. This is caused by an increased expression of ABA synthesis and GA degradation enzyme genes (Figure 2). A higher expression of genes related to ABA sensitivity is also associated with high seed dormancy levels (Footitt et al., 2011) (Figure 2). In contrast, imbibed seeds with low dormancy show an increased expression of ABA degradation and GA synthesis enzyme genes, leading to a reduction in ABA and a rise of active GA accompanied by a higher expression of GA sensitivity genes (Finch-Savage and Footitt, 2017) (Figure 2).

Several genes were recently identified as strongly related to physiological dormancy, such as *DELAY OF GERMINATION 1* (*DOG1*), *SEED DORMANCY 4* (*SDR4*), and *MOTHER OF FT AND TFL 1* (*MFT*). All of them were discovered by QTL analysis using variable natural and synthetic populations of *Arabidopsis*, rice and wheat. (Sugimoto et al., 2010; Nakamura et al., 2011; Carrillo-Barral et al., 2020). High amounts of transcripts and proteins from such dormancy-specific genes were found in seeds of highly dormant genotypes or under environmental conditions leading to high seed dormancy expression (Finch-Savage and Footitt, 2017; Carrillo-Barral *et al.*, 2020). Although our understanding of the function of these dormancy-specific genes is far from complete, evidence suggests that these genes may link the environmental conditions during seed development or after dispersion with ABA sensitivity and seed dormancy levels (Footitt *et al.*, 2011) (Figure 2). 

Genes associated with chromatin restructuring, such as *HISTONE MONOUBIQUITYLATION-1* (*HUB1*) and *REDUCED DORMANCY 2* (*RDO2*), are also involved in seed dormancy regulation. Mutant plants with altered acetylation, methylation and ubiquitination of histones show differences in seed dormancy phenotypes compared with wild-type plants (Carrera-Castaño et al., 2020). Additionally, changes in activation or repression of chromatin condensation in genome regions where seed dormancy genes are located affect the level of seed dormancy (Finch-Savage and Footitt, 2017; Carrera-Castaño *et al.*, 2020). These epigenetic mechanisms may explain part of the phenotypic plasticity of dormancy caused by maternal effects.

## Crop domestication and seed dormancy

Domestication is the result of artificial selection process on a variable plant population, altering traits that make them suitable for cultivation (Fuller and Allaby, 2009). These selections may have been gradual processes over hundreds or thousands of years, although some key domestication traits are controlled by a single gene (Glémin and Bataillon, 2009), and changes are immediately observed. Natural genetic variation has always been the starting point of any domestication process. Low mutation rates suggest that the ‘domestication genes’ and genetic variation within crops were components of the ancestral wild gene pool (Glémin and Bataillon, 2009). 

The set of traits related to domestication, referred to as domestication syndrome, is often similar across different species (Fuller and Allaby, 2009). These traits are the product of selecting the main objectives of crop cultivation, such as increasing seed harvesting, yield, and seedling competition (Glémin and Bataillon, 2009). It is not surprising that several traits of this syndrome are seed-related. Retention of seeds on mother plants is one of the most relevant traits because it increases the chances of successful seed harvesting by humans. Another relevant trait is the larger size of crop seeds compared to their wild relatives which increases seed yield and improves seedling vigor (Fuller and Allaby, 2009). A less evident but not less important seed-related trait is dormancy.

## Domesticated crops have very low seed dormancy

In wild populations, seed dormancy allows the dispersion of progeny over time, and it is considered an adaptation to fluctuating environments. Seed populations germinate mostly in pulses and a fraction may remain in the soil waiting for better conditions for seedling growth (Donohue et al., 2010). This behavior is disadvantageous in agroecosystems where the environment is more predictable and competition is favored. A shallow level of seed dormancy coupled with low intrapopulation variability facilitates rapid and synchronized seed germination in the field under a wide range of environmental conditions, which is highly desirable for crop establishment (Mitchell et al., 2017). Thus, very low seed dormancy is another component of the domestication syndrome, and it is a convergent phenotype among crops. Evidence shows that seed dormancy genes have been subject to parallel selection in various crop families, including Poaceae, Fabaceae and Brassicaceae (Wang et al., 2018). Nevertheless, an excessively low level of seed dormancy may also enable seed germination on the mother plant, known as pre-harvest sprouting, a severe issue in modern cereal cultivars (Rodríguez et al., 2015). A significant part of what we know about seed dormancy is based on solving this unintended consequence of domestication. 

Although it is desirable for agricultural purposes, selection for reduced seed dormancy was largely unconscious, much like other domestication traits (Glémin and Bataillon, 2009). In the early stages of domestication, ancient farmers harvested seeds from the fraction of the crop population that could germinate, indirectly selecting low-dormancy genotypes (Fuller and Allaby, 2009). Additionally, the preference for lighter cereal caryopsis coat coloration, possibly due to intentional selection for reduced toxicity and increased grain palatability, may have indirectly contributed to the reduction of seed dormancy. This relationship arises because, in general, cereal genotypes with lighter caryopsis coats show lower levels of phenolic compounds and polyphenol oxidase activity. These components of phenolic metabolism are associated with ABA metabolism, leading to the maintenance of seed dormancy (Rodríguez et al., 2015). 

## Seed dormancy in semi-domesticated crops: forage grasses

Grass species used for forage or biomass production are mostly in a less advanced state of domestication than cereal crops. While many forage cultivars show noticeable differences from their wild relatives in forage production, forage quality, and stress tolerance, they do not show significant differences in seed traits associated with the domestication syndrome, such as seed retention, size or dormancy (Wilkins and Humphreys, 2003; Pereira et al., 2018). 

Several non-mutually exclusive factors have been proposed to explain this semi-domestication status of forage crops: i) the beginning of the domestication process in forage crops is much more recent (Wilkins and Humphreys, 2003); ii) breeding of improved varieties has focused on vegetative and phenological traits associated with forage production, palatability, and grazing behavior rather than on seed traits; iii) grass species used for forage production often have breeding barriers and limitations. Some species exhibit self-incompatibility, which limits allele fixation due to outcrossing. Other species exhibit apomixis, which ensures allele fixation but hinders genetic variability and gene segregation relevant for breeding (Casler, 2012; Pereira et al., 2018). 

Forage production in temperate climates is dominated by ryegrass (*Lolium* spp.) and fescues (*Festuca* spp.). While breeding efforts for these species pose no significant constraints, they were not focused on seed traits except for some efforts to reduce seed shattering (Wilkins and Humphreys, 2003). However, ryegrass and fescue cultivars often exhibit a relatively low level of seed dormancy, which decreases significantly within a few months after harvesting, leading to a good seedling emergence and field establishment in a broad environmental range (Steadman, 2004; Ahmed and Escobar-Gutiérrez, 2022). Some evidence suggests no significant differences in seed dormancy between cultivars and their wild relatives (Ghaleb et al., 2022). Interestingly, it has been suggested that the low seed dormancy found in grass species abundant in European meadows may have resulted from unconscious selective pressure exerted by winter hay and livestock management practiced during the Middle Ages in Europe (Harlan, 1992). 

## Seed dormancy in warm-season forage grasses

In contrast to ryegrass or fescues, many forage cultivars of warm-season grasses show remarkably high seed dormancy levels. Because most of these species have been recently introduced into cultivation, similarly to other domestication traits, they do not show differences in seed dormancy between cultivars and their wild relatives (Adkins et al., 2002). This high level of dormancy increases the risk of failure of crop establishment, which is one of the primary reasons why the adoption of warm-season grasses is particularly challenging, except in specific locations (McCormick et al., 2009; Kimura et al., 2015; Pereira et al., 2018). Reducing seed dormancy to levels comparable to those of widely adopted cool-season forage crops should be a goal to improve the commercial adoption of warm-season grasses. 

## Management adaptations to seed dormancy for cultivating warm-season grasses

Warm-season grasses exhibit high seed dormancy, which means they can only germinate under specific environmental conditions. Typically, these conditions include warm to high temperatures, daily fluctuations in temperature, light, and sufficient water availability (Adkins et al., 2002; Kimura et al., 2015). These restrictions in germination conditions require selecting the optimal sowing time based on the availability of adequate rainfall, either in the spring or summer, resulting in variable sowing dates across locations (Masters et al., 2004). In addition, weed control and a shallow sowing depth are recommended to achieve a higher seedling emergence of warm-season grasses (Lodge and Harden, 2009). These recommendations help to meet the requirements for dormancy-breaking factors such as alternating temperatures, light and nitrate availability (Adkins *et al.*, 2002; Batlla and Benech-Arnold, 2010; Kimura *et al.*, 2015).

Farmers cultivating warm-season grasses for forage have adapted their seed management and sowing timing to avoid or reduce seed dormancy and achieve higher crop establishment rates. One approach involves reducing primary seed dormancy by storing them in a dry environment for at least a year, resulting in seeds with higher germinability (Kimura et al., 2015). Another method proposed is sowing the seeds during the dormant season (winter and early spring) to subject them to cold stratification before the growing season begins, effectively reducing seed dormancy (Keyser et al., 2016). Alternative methods to reduce seed dormancy before sowing have been explored. These include seed priming (pre-germination moistening), the addition of phytohormones or biological agents, and scarification treatments (Schrauf et al., 1995; Kimura *et al.*, 2015; de Agostini et al., 2022). Unfortunately, the available methods do not consistently provide reliable results or may not be suitable for commercial procedures.

## Breeding warm-season grasses for reduced seed dormancy

Current methods for managing high seed dormancy in warm-season forage grasses have not been sufficient to facilitate their use. Breeding efforts aimed at reducing seed dormancy are the most effective solution. To accomplish this, a wide range of phenotypic variability should be available first. Switchgrass (*Panicum virgatum* L.), for example, is a native perennial species from North America that has been bred as a forage and biomass crop (Casler, 2012). In species with sexual reproduction, such as switchgrass, intraspecific variability in seed dormancy is very wide (Seepaul et al., 2011), so switchgrass cultivars were selected from the natural variation and further improvements in forage production were achieved (Casler, 2012). Although not specifically bred for this purpose, it has been observed that switchgrass cultivars show a lower seed dormancy level than wild populations on average (Eckberg et al., 2015). New cultivars and experimental lines with reduced seed dormancy were developed in a few generations by selecting the population fraction capable of germinating earlier or under low water potential in switchgrass, bahiagrass (*Paspalum notatum* Flügge) and sand bluestem (*Andropogon hallii* Hack.). Selected cultivars with low seed dormancy can achieve a higher field establishment (Burson et al., 2009; Anderson et al., 2011; Springer et al., 2012). 

Apomixis is particularly prevalent among warm-season grasses cultivated in tropical climates. Near 50 M ha of forage production in Brazil are cultivated with a single tetraploid apomictic clone, *Urochloa brizantha* cv. Marandú (Pereira et al., 2018). Apomictic reproduction is desirable for crops because it produces seeds that retain hybrid vigor and high heterozygosity across generations. However, apomixis strongly hinders breeding efforts in several species (Pereira *et al.*, 2018; Acuña et al., 2019; Monteverde et al., 2022). Nevertheless, unexpected intraspecific genetic variation can be found in these species. Apomictic clones are often part of polyploid species complexes, often occurring with closely related sexual germplasm. In these locations, recombinant apomictic clones may appear (Speranza, 2009; Acuña *et al.*, 2019) and apomictic clones with lower than average seed dormancy levels can be found (Glison et al., 2015). Efforts to use sexual materials to release the variability contained in the sexual and apomictic gene pool have been made; however, the efficiency of generating such hybrids remains limited (Miles et al., 2006; Novo et al., 2017).

Molecular technologies can assist breeding in warm-season grasses. Until recently, the identification of molecular markers and sequenced genomes were only available for a small group of warm-season forage grass species, such as switchgrass, signalgrass (*Urochloa* spp.) and foxtail grass (*Setaria* spp.) (Casler, 2012; Hu et al., 2018; Pereira et al., 2018). Currently, genotyping-by-sequencing (GBS) technologies can be applied in non-model species without a reference genome (Elshire et al., 2011). This technology and other molecular tools were used to increase the number of warm-season grasses with a sequenced genome, mapped molecular markers and transcriptome analyses (Wang et al., 2012; Hu et al., 2018; Pereira et al., 2018; Cui et al., 2021). These genomic tools were used to identify genes aimed at facilitating assisted breeding for forage traits, such as abiotic and biotic stress tolerance, forage nutritional value and yield, or flowering date (Hu et al., 2018; Pereira et al., 2018; Razar and Missaoui, 2020). However, genomic studies for germination and seed dormancy traits are notably lacking despite the importance of these traits for the effective domestication of these species.

## Advances in the domestication of *Paspalum dilatatum* focusing on seed dormancy

Selecting early domesticates with lower seed dormancy levels is crucial to improve seedling emergence and crop establishment (Mitchell et al., 2015). In this section, we describe advances in the domestication of dallisgrass (*Paspalum dilatatum* Poir.) and its related species as a case study of an ongoing domestication process that prioritized seed dormancy.

*Paspalum dilatatum* var. *dilatatum* is a warm-season grass taxon comprising an array of pentaploid apomictic clones. These clones show a remarkable forage aptitude and can tolerate the most frequent abiotic stresses in the Campos biome, including frost, drought, and floods (Loreti and Oesterheld, 1996; Campbell et al., 1999). Its forage aptitude was recognized over a century ago when it was introduced to sown pastures in Australia and New Zealand in 1880, triggering a revolution in forage production (Star and Brooking, 2006). Since then, these clones have been introduced in multiple countries and have established naturalized populations in those regions. 

Most dallisgrass cultivars are apomictic pentaploids that were chosen from wild accessions (Acuña et al., 2019). Although important advances have been made in our understanding of the phenology of flowering and seed production dynamics in the species (González Barrios et al., 2016; Casalás et al., 2023), the available cultivars do not represent any specific advances in traits related to seed dormancy which restrict its productive adoption. Several efforts were made to increase genetic variability, such as hybridization, mutagenesis and transgenesis (Vaio et al., 2007; Giordano et al., 2014), but none has resulted in a released cultivar. The presence of apomixis has been a significant obstacle to dallisgrass domestication.

## The nature of seed dormancy in dallisgrass

Seed dormancy in dallisgrass shows similar characteristics to those of other warm-season grasses. Warm to hot temperatures and dark-light daily alternation (20 °C in the dark, 30 or 35 °C with light), as well as adding a nitrate solution (0.2% KNO_3_), are recommended to achieve high seed germination proportion (ISTA, 2015). Seed dormancy is mostly expressed at lower temperatures or when dormancy-breaking factors (light, nitrate, alternating temperatures) are absent. Freshly harvested seeds have a high level of dormancy that can be significantly reduced after six months of dry storage or one to two weeks of stratification (Schrauf et al., 1995; Glison et al., 2015). However, the effectiveness of these methods may be conditioned by temperature and seed moisture level. The requirements to break dormancy depend on the genotype and show slight differences among a broad and variable set of pentaploid apomictic clones (Glison *et al.*, 2015). 

Seed scarification treatments result in increased germination proportions. The caryopsis of dallisgrass seeds is tightly and fully enclosed by coriaceous lemma and palea. The removal of such floral structures drastically relieves seed dormancy, and the seeds can germinate in colder temperatures without dormancy-breaking factors (Glison et al., 2017). Such evidences supported the hypothesis that the impediment of water uptake was the main cause of seed dormancy (physical dormancy). In spite of this, it has been shown that imbibition is not impeded in non-scarified seeds, and the mechanisms of coat-based dormancy in dallisgrass are physiological and related to ABA sensitivity (Glison *et al.*, 2017).

## Genetic variability and breeding for reduced seed dormancy

**Figure 3 -:**
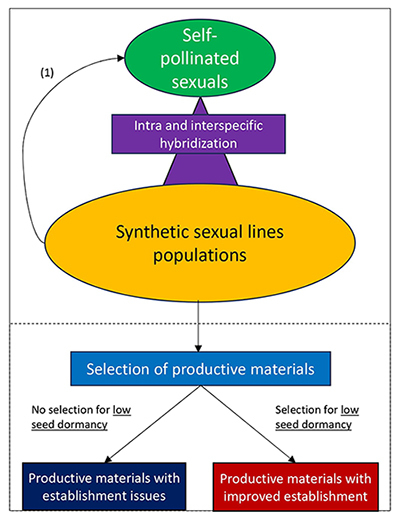
Representation of the suggested strategy to obtain warm-season forage grasses adapted to temperate regions using sexual *Paspalum* species from the Dilatata group. The ovals represent potential genetic pools, and their sizes represent the magnitude of the genetic variability. Natural genetic variability includes the five species along with their intraspecific variability (green oval). The genetic variability can be substantially increased by inter and intraspecific hybridizations that generate synthetic populations (orange oval), and it may be broader due to instances of hybridization between synthetic and natural lines (1). After developing a population showing genetic variability, the next task is to select the most adapted and productive forage material (light blue rectangle). However, if choosing for lower seed dormancy is not considered (left), the risk of selecting good forage materials with crop establishment issues increases (blue rectangle). Screening for low seed dormancy is suggested (right) to definitively reach a material with higher propagation efficiency from seeds in the agricultural landscapes (red rectangle).

Pentaploid *P. dilatatum* belongs to an allopolyploid species complex (Dilatata complex) that also encompasses other apomictic penta- hexa- and heptaploids, as well as five sexual tetraploid species (Speranza, 2009; Rosso et al., 2022). Efforts were first directed toward exploring phenotypic variability among wild accessions. The aim was to find materials that can overcome establishment and production limitations, including seed dormancy (Speranza *et al.*, 2017). During this screening process, a hexaploid apomictic material (*P. dilatatum* var. Chirú) displayed notably low seed dormancy compared to other apomictic materials (Glison et al., 2015, 2017). Also, differences in seed dormancy were evident between two of the sexual species. *Paspalum flavescens* (formerly *P. dilatatum* subsp. *flavescens*) shows higher seed dormancy than *P. plurinerve* (formerly *P. dilatatum* biotype Virasoro) (Glison *et al.*, 2015, 2017; Rosso *et al.*, 2022). Thus, sexual species became the target for finding genetic variability and differences in seed dormancy within the Dilatata group (Figure 3). 

The importance of seed dormancy levels was explored in relation to the timing and extent of seedling emergence in field conditions with and without irrigation across various years, locations, and sowing dates. Seedling emergence in *P. dilatatum* var. Chirú (low seed dormancy) was faster and achieved higher proportions than *P. flavescens* (high seed dormancy) in most trials, mainly in non-irrigated conditions (Glison et al., 2021a ), suggesting a lower seed dormancy level may contribute to a better crop establishment rate in this group of species. In addition, early spring sowings resulted in higher seedling emergence than late spring sowings, even in situations with high water availability in the soil. These results indicate that early spring is the optimum time to sow dallisgrass materials, and high temperatures during late spring may increase seed dormancy (Glison *et al.*, 2021a).

The native ranges of the sexual Dilatata species are in the midlatitude lowland regions of South America. Three of them (*P. flavescens*, *P. plurinerve*, and *P. vacarianum*), which show plant morphology and forage potential similar to dallisgrass, have restricted and allopatric distribution along the Campos biome (Rosso et al., 2022). There are no evident differences in vegetative functional traits among these species, although very significant differences are observed in seed dormancy. A clear pattern of decreasing seed dormancy levels was recorded among these sexual species, which correlated with increasing rainfall regimes across their distribution area (Glison et al., 2023). The evidence suggests that regeneration traits, particularly differences in seed dormancy, explain their allopatric distribution, and there may be a wide diversity of alleles for the genes that define this trait among these species. 

Despite their genetic differences and highly autogamous behavior, the sexual species of the Dilatata group are phylogenetically very closely related (Vaio et al., 2019), and vigorous hybrids can be generated through interspecific crossing in certain combinations (Figure 3). Recombinant F3 families obtained from a single F1 hybrid by crossing *P. flavescens* and *P. plurinerve* showed potential for transgressive segregation in several vegetative traits, indicating that this breeding strategy is highly promising (Monteverde et al., 2022). As part of the ongoing research, a recombinant inbred line (RIL) population from a *P. flavescens* × *P. plurinerve* F1 hybrid was obtained to conduct a QTL analysis for various traits, including seed dormancy, on a SNP-based genetic map. High variability in seed dormancy was found among lines, and transgressive segregation for seed dormancy was observed. Notably, this trait showed high heritability in this population. Two QTL with significant effects on seed dormancy were detected in two different linkage groups, suggesting independent control of this trait (Glison et al., 2021b ). Collectively, these results suggest the high potential of using sexual species within a conventional breeding program to develop new dallisgrass cultigens. However, it is essential to consider low seed dormancy levels in the selection process to overcome the limitations that this trait poses during crop establishment (Figure 3).

## Conclusions

Low seed dormancy is a common trait found in domesticated plants, including some forage crops. However, breeding programs for warm-season forage grasses have largely overlooked this trait. Although current knowledge of the physiological and genetic mechanisms of seed dormancy in warm-season forage grasses is lacking, we know that these mechanisms are highly conserved among species with physiological dormancy, and valuable lessons can be learned from cereal crops. The increased availability of advanced genomic tools may facilitate the transfer of such knowledge to novel species. We walked through the initial stages of the *de novo* domestication process of a warm-season grass by describing the case of dallisgrass. The primary objectives during the domestication process should be to understand genetic variability and heritability patterns within the available gene pool. We emphasize the importance of seed dormancy because the inability to germinate makes seeds unsuitable for commercial adoption of the species. Currently, we have gathered a significant amount of information on seed dormancy within the Dilatata group, and the first genomic tools for these species were developed. Many authors propose crop diversification by domesticating species to increase agroecosystem sustainability and resilience to future climate issues. We claim that not only traits directly associated with productive aptitude should be emphasized when a new forage species is to be brought under cultivation. Instead, the modification of other domestication traits, such as seed dormancy, deserves special attention.

## References

[B1] Acuña CA, Martínez EJ, Zilli AL, Brugnoli EA, Espinoza F, Marcón F, Urbani MH, Quarin CL (2019). Reproductive systems in Paspalum: Relevance for germplasm collection and conservation, breeding techniques, and adoption of released cultivars. Front Plant Sci.

[B2] Adkins SW, Bellairs SM, Loch DS (2002). Seed dormancy mechanisms in warm season grass species. Euphytica.

[B3] Ahmed LQ, Escobar-Gutiérrez AJ (2022). Unexpected intraspecific variability of perennial ryegrass (Lolium perenne L.) in response to constant temperature during germination and initial heterotrophic growth. Front Plant Sci.

[B4] Anderson WF, Gates RN, Hanna WW (2011). Registration of ‘TifQuik’ bahiagrass. J Plant Regist.

[B5] Batlla D, Benech-Arnold RL (2010). Predicting changes in dormancy level in natural seed soil banks. Plant Mol Biol.

[B6] Benech-Arnold RL, Sánchez RA, Forcella F, Kruk BC, Ghersa CM (2000). Environmental control of dormancy in weed seed banks in soil. Field Crops Res.

[B7] Bentsink L, Hanson J, Hanhart CJ, Blankestijn-de Vries H, Coltrane C, Keizer P, El-Lithy M, Alonso-Blanco C, de Andrés MT, Reymond M (2010). Natural variation for seed dormancy in Arabidopsis is regulated by additive genetic and molecular pathways. Proc Natl Acad Sci U S A.

[B8] Burson BL, Tischler CR, Ocumpaugh WR (2009). Breeding for reduced post-harvest seed dormancy in switchgrass: Registration of TEM-LoDorm switchgrass germplasm. J Plant Regist.

[B9] Campbell BD, Mitchell ND, Field TRO (1999). Climate profiles of temperate C3 and subtropical C4 species in New Zealand pastures. New Zeal J Agric Res.

[B10] Carrera-Castaño G, Calleja-Cabrera J, Pernas M, Gómez L, Oñate-Sánchez L (2020). An updated overview on the regulation of seed germination. Plants (Basel).

[B11] Carrillo-Barral N, Rodríguez-Gacio M del C, Matilla AJ (2020). Delay of germination-1 (DOG1): A key to understanding seed dormancy. Plants (Basel).

[B12] Casalás F, Speranza PR, Cadenazzi M, Zanoniani R, Giménez L, Boggiano P (2023). Effect of irrigation on biomass production and components of dallisgrass (Paspalum dilatatum) and bahiagrass (P. notatum) in Uruguay. Trop Grasslands.

[B13] Casler MD, Monti A (2012). Switchgrass, green energy and technology.

[B14] Cui F, Taier G, Li M, Dai X, Hang N, Zhang X, Wang X, Wang K (2021). The genome of the warm-season turfgrass African bermudagrass (Cynodon transvaalensis). Hortic Res.

[B15] de Agostini RT, Abrantes FL, Machado NB, Custódio CC (2022). Ethanol and hormones in physiological conditioning on germination and seed dormancy of Urochloa humidicola cv. Llanero. J Seed Sci.

[B16] Donohue K, Rubio de Casas R, Burghardt L, Kovach K, Willis CG (2010). Germination, postgermination adaptation, and species ecological ranges. Annu Rev Ecol Evol Syst.

[B17] Eckberg JO, Casler MD, Johnson GA, Seefeldt LL, Blaedow KE, Shaw RG (2015). Switchgrass population and cold-moist stratification mediate germination. Crop Sci.

[B18] Elshire RJ, Glaubitz JC, Sun Q, Poland JA, Kawamoto K, Buckler ES, Mitchell SE (2011). A robust, simple genotyping-by-sequencing (GBS) approach for high diversity species. PLoS One.

[B19] Finch-Savage WE, Footitt S (2017). Seed dormancy cycling and the regulation of dormancy mechanisms to time germination in variable field environments. J Exp Bot.

[B20] Footitt S, Douterelo-Soler I, Clay H, Finch-Savage WE (2011). Dormancy cycling in Arabidopsis seeds is controlled by seasonally distinct hormone-signaling pathways. Proc Natl Acad Sci U S A.

[B21] Fuller DQ, Allaby R, Ostergaard L (2009). Fruit development and seed dispersal.

[B22] Ghaleb W, Barre P, Teulat B, Ahmed LQ, Escobar-Gutiérrez AJ (2022). Divergent selection for seed ability to germinate at extreme temperatures in perennial ryegrass (Lolium perenne L.). Front Plant Sci.

[B23] Giordano A, Liu Z, Panter SN, Dimech AM, Shang Y, Wijesinghe H, Fulgueras K, Ran Y, Mouradov A, Rochfort S (2014). Reduced lignin content and altered lignin composition in the warm season forage grass Paspalum dilatatum by down-regulation of a Cinnamoyl CoA Reductase gene. Transgenic Res.

[B24] Glémin S, Bataillon T (2009). A comparative view of the evolution of grasses under domestication: Tansley review. New Phytol.

[B25] Glison N, Viega L, Cornaglia P, Gutiérrez L, Speranza PR (2015). Variability in germination behaviour of Paspalum dilatatum Poir. seeds is genotype dependent. Grass Forage Sci.

[B26] Glison N, Viega L, Speranza PR (2017). Differential incidence of the lemma on seed germination among different Paspalum dilatatum genotypes. J Seed Sci.

[B27] Glison N, Batlla D, González Barrios P, Viega L, Saldanha S, Musacchio EM, Rush P, Speranza PR (2021). Modelling seedling emergence in Paspalum species using environmental data from field experiments. Grass Forage Sci.

[B28] Glison N, Monteverde E, Speranza PR (2021). Análisis de QTL para la dormición de semillas en una población de líneas recombinantes de Paspalum grupo Dilatata. J Basic Appl Genet.

[B29] Glison N, Romero D, Rosso V, Guerrero JC, Speranza PR (2023). Understanding the geographic patterns of closely-related species of Paspalum (Poaceae) using distribution modelling and seed germination traits. Plants (Basel).

[B30] González Barrios P, Speranza PR, Glison N, Piccardi M, Balzarini M, Gutierrez L (2016). Analysis of flowering dynamics heritability in the perennial warm‐season grass Paspalum dilatatum. Grass Forage Sci.

[B31] Graeber K, Nakabayashi K, Miatton E, Leubner-Metzger G, Soppe WJJ (2012). Molecular mechanisms of seed dormancy. Plant Cell Environ.

[B32] Harlan JR, Chapman GP (1992). Grass evolution domestication.

[B33] Hu H, Mauro-Herrera M, Doust AN (2018). Domestication and improvement in the model C4 grass, Setaria. Front Plant Sci.

[B34] ISTA - International Seed Testing Association (2015). International rules for seed testing.

[B35] Keyser PD, Ashworth AJ, Allen FL, Bates GE (2016). Dormant-season planting and seed-dormancy impacts on switchgrass establishment and yield. Crop Sci.

[B36] Kimura E, Fransen SC, Collins HP, Guy SO, Johnston WJ (2015). Breaking seed dormancy of switchgrass (Panicum virgatum L.): A review. Biomass Bioenergy.

[B37] Lodge GM, Harden S (2009). Effects of depth and time of sowing and over-wintering on tropical perennial grass seedling emergence in northern New South Wales. Crop Pasture Sci.

[B38] Loreti J, Oesterheld M (1996). Intraspecific variation in the resistance to flooding and drought in populations of Paspalum dilatatum from different topographic positions. Oecologia.

[B39] Malavert C, Batlla D, Benech-Arnold RL (2021). The role of seed water content for the perception of temperature signals that drive dormancy changes in Polygonum aviculare buried seeds. Funct Plant Biol.

[B40] Masters RA, Mislevy P, Moser LE, Rivas-Pantoja F, Moser LE, Burson BL, Sollenberger LE (2004). Warm-Season (C4) Grasses.

[B41] McCormick LH, Boschma SP, Lodge GM, Scott JF (2009). Producer-identified constraints to widespread adoption of sown tropical grass pastures on the north-west slopes of New South Wales. Trop Grasslands.

[B42] Miles JW, Cardona C, Sotelo G (2006). Recurrent selection in a synthetic brachiariagrass population improves resistance to three spittlebug species. Crop Sci.

[B43] Mitchell J, Johnston IG, Bassel GW (2017). Variability in seeds: Biological, ecological, and agricultural implications. J Exp Bot.

[B44] Mitchell ML, Norman HC, Whalley RDB (2015). Use of functional traits to identify Australian forage grasses, legumes and shrubs for domestication and use in pastoral areas under a changing climate. Crop Pasture Sci.

[B45] Monteverde E, Olveyra M, Speranza PR (2022). Could the Dilatata group of Paspalum be bred as sexual species? A preliminary assessment. Grass Forage Sci.

[B46] Nakamura S, Abe F, Kawahigashi H, Nakazono K, Tagiri A, Matsumoto T, Utsugi S, Ogawa T, Handa H, Ishida H (2011). A wheat homolog of MOTHER OF FT AND TFL 1 acts in the regulation of germination. Plant Cell.

[B47] Novo PE, Acuña CA, Quarin CL, Urbani MH, Marcón F, Espinoza F (2017). Hybridization and heterosis in the Plicatula group of Paspalum. Euphytica.

[B48] Pereira JF, Azevedo ALS, Pessoa M, Romanel EAC, Pereira AV, Vigna BBZ, de Souza F, Benites FRG, Lédo FJS, Brito GG (2018). Research priorities for next-generation breeding of tropical forages in Brazil. Crop Breed Appl Technol.

[B49] Razar RM, Missaoui A (2020). QTL mapping of winter dormancy and associated traits in two switchgrass pseudo-F1 populations: Lowland x lowland and lowland x upland. BMC Plant Biol.

[B50] Rodríguez MV, Barrero JM, Corbineau F, Gubler F, Benech-Arnold RL (2015). Dormancy in cereals (not too much, not so little): About the mechanisms behind this trait. Seed Sci Res.

[B51] Rosso VC, Valls JFM, Quarin CL, Speranza PR, Rua GH (2022). New entities of Paspalum and a synopsis of the group Dilatata. Syst Bot.

[B52] Schrauf GE, Cornaglia PS, Deregibus VA, Ríssola MG (1995). Improvement in germination behaviour of Paspalum dilatatum Poir. seeds under different pre‐conditioning treatments. New Zeal J Agric Res.

[B53] Seepaul R, Macoon B, Reddy KR, Baldwin B (2011). Switchgrass (Panicum virgatum L.) intraspecific variation and thermotolerance classification using in vitro seed germination assay. Am J Plant Sci.

[B54] Speranza PR (2009). Evolutionary patterns in the Dilatata group (Paspalum, Poaceae). Plant Syst Evol.

[B55] Speranza PR, Viega L, Gutierrez L, Astigarraga L, Picasso V, Saldanha S, Glison N, Gonzalez Barrios P, Sandro P, Tejera M (2017). Utilización y domesticación de gramíneas forrajeras del género Paspalum en Uruguay. Revista INIA Uruguay.

[B56] Springer TL, Wynia RL, Rea GL (2012). Field emergence and plant density of sand bluestem lines selected for increased seed germination. Crop Sci.

[B57] Star P, Brooking T (2006). Fescue to the rescue: Chewings fescue, Paspalum, and the application of non-British experience to pastoral practice in New Zealand, 1880-1920. Agric Hist.

[B58] Steadman KJ (2004). Dormancy release during hydrated storage in Lolium rigidum seeds is dependent on temperature, light quality, and hydration status. J Exp Bot.

[B59] Subburaj S, Cao S, Xia X, He Z (2016). Phylogenetic analysis, lineage-specific expansion and functional divergence of seed dormancy 4-like genes in plants. PLoS One.

[B60] Sugimoto K, Takeuchi Y, Ebana K, Miyao A, Hirochika H, Hara N, Ishiyama K, Kobayashi M, Ban Y, Hattori T (2010). Molecular cloning of Sdr4, a regulator involved in seed dormancy and domestication of rice. Proc Natl Acad Sci U S A.

[B61] Vaio M, Mazzella C, Porro V, Speranza PR, López-Carro B, Estramil E, Folle GA (2007). Nuclear DNA content in allopolyploid species and synthetic hybrids in the grass genus Paspalum. Plant Syst Evol.

[B62] Vaio M, Mazzella C, Guerra M, Speranza PR (2019). Effects of the diploidisation process upon the 5S and 35S rDNA sequences in the allopolyploid species of the Dilatata group of Paspalum (Poaceae, Paniceae). Aust J Bot.

[B63] Van Tassel D, De Haan L (2013). Wild plants to the rescue. Am Sci.

[B64] Wang M, Li W, Fang C, Xu F, Liu Y, Wang Z, Yang R, Zhang M, Liu S, Lu S (2018). Parallel selection on a dormancy gene during domestication of crops from multiple families. Nat Genet.

[B65] Wang Y, Zeng X, Iyer NJ, Bryant DW, Mockler TC, Mahalingam R (2012). Exploring the switchgrass transcriptome using second-generation sequencing technology. PLoS One.

[B66] Wilkins PW, Humphreys MO (2003). Progress in breeding perennial forage grasses for temperate agriculture. J Agric Sci.

